# Psychological functioning in adolescents referred to specialist gender identity clinics across Europe: a clinical comparison study between four clinics

**DOI:** 10.1007/s00787-017-1098-4

**Published:** 2017-12-18

**Authors:** Nastasja M. de Graaf, Peggy T. Cohen-Kettenis, Polly Carmichael, Annelou L. C. de Vries, Karlien Dhondt, Jolien Laridaen, Dagmar Pauli, Juliane Ball, Thomas D. Steensma

**Affiliations:** 1 Gender Identity Development Service, Tavistock and Portman NHS Foundation Trust, 120 Belsize Lane, London, NW3 5BA UK; 2 Department of Medical Psychology, Center of Expertise on Gender Dysphoria, VU University Medical Center, Amsterdam, The Netherlands; 3 Department of Child and Adolescent Psychiatry, Center of Expertise on Gender Dysphoria, VU University Medical Center, Amsterdam, The Netherlands; 4 Pediatric Gender Clinic, Center for Sexuology and Gender, Ghent University Hospital, Ghent, Belgium; 5 Department of Child- and Adolescent Psychiatry, University Hospital of Psychiatry, Zurich, Switzerland

**Keywords:** Gender identity, Gender diverse adolescents, Psychological functioning, Behavioural problems, Peer relations

## Abstract

Adolescents seeking professional help with their gender identity development often present with psychological difficulties. Existing literature on psychological functioning of gender diverse young people is limited and mostly bound to national chart reviews. This study examined the prevalence of psychological functioning and peer relationship problems in adolescents across four European specialist gender services (The Netherlands, Belgium, the UK, and Switzerland), using the Child Behavioural Checklist (CBCL) and the Youth Self-Report (YSR). Differences in psychological functioning and peer relationships were found in gender diverse adolescents across Europe. Overall, emotional and behavioural problems and peer relationship problems were most prevalent in adolescents from the UK, followed by Switzerland and Belgium. The least behavioural and emotional problems and peer relationship problems were reported by adolescents from The Netherlands. Across the four clinics, a similar pattern of gender differences was found. Birth-assigned girls showed more behavioural problems and externalising problems in the clinical range, as reported by their parents. According to self-report, internalising problems in the clinical range were more prevalent in adolescent birth-assigned boys. More research is needed to gain a better understanding of the difference in clinical presentations in gender diverse adolescents and to investigate what contextual factors that may contribute to this.

## Introduction

Growing awareness of specialist gender identity clinics and acceptance of gender diversity by the wider community may have contributed to the increasing number of children and adolescents seeking professional help with their gender identity development [1]. Some young people presenting to specialist gender identity clinics may question or struggle with societal gender stereotypes. Others may seek recognition of their gender diverse feelings and wish to attain a body that is congruent with their experienced gender identity [2]. In this paper, young people who request help with a felt incongruence between their gender identity and the gender to which they were assigned at birth are referred to as gender diverse young people. Many of those, but not all, would meet the diagnostic criteria for gender dysphoria (GD) [3]. The initial assessment phase is aimed at understanding the young person’s development and gender identification in the context of their family background and life experiences.

Gender diversity is frequently associated with psychological difficulties. Existing literature on psychological functioning in gender diverse adolescents shows greater prevalence of associated difficulties as compared to the general population. At the specialist gender identity clinic in London, bullying (47%), low mood (42%), and self-harming thoughts and behaviours (39%) were the three most common associated difficulties observed [4–6]. In The Netherlands 32.4% of the adolescents referred to the Amsterdam clinic had a co-occurring psychiatric disorder, most commonly anxiety disorder (21%) [7]. Higher rates of psychiatric comorbidity were also mentioned in Finland, revealing that 64% of the gender diverse adolescents had received treatment due to depression, 55% due to anxiety, and 53% due to suicidal and self-harming behaviours [8].

These findings are not solely found in European countries. In the USA, high prevalence of depression, suicidal thoughts and attempts, and substance abuse were reported by several independent gender clinics across the nation [9–12]. In a study from Spack et al. [13], 44.3% of gender diverse adolescents presented with a history of psychiatric diagnoses. Grossman and D’Augelli [14] reported that 45% of transgender youth had suicidal thoughts and 26% reported a history of life-threatening behaviours.

As with the studies above, most of the published evidence on gender diverse adolescents has been based on independent clinical chart reviews. The only direct comparison studies in psychological functioning in gender diverse youth was conducted by The Netherlands and North-American specialist gender identity clinics, using standardised parent- and self-report measures such as the Child Behavioural Checklist (CBCL) and the Youth Self-Report (YSR) [15, 16]. In these studies, 40.6% of adolescents from The Netherlands and 39.9% of Canadian adolescents reported significant behavioural and emotional problems in the clinical range on the YSR [17]. They found a predominance of internalising problems over externalising problems. On average, emotional and behavioural problems in the clinical range were more prevalent in Canada compared to The Netherlands [11, 17, 18].

Poor peer relationships were found to be the strongest predictor for emotional and behavioural problem scores in gender diverse youth [11, 17, 18]. Social ostracism has been suggested to be an important risk factor that contributes to increased psychological distress in gender diverse adolescents [17–20]. Likewise, in sexual minorities, factors of social ostracism (such as peer exclusion or peer rejection) were linked to an elevated risk of self-harming thoughts and behaviours [21–23]. It is still unclear to what extent social ostracism influences the presence of psychopathology in gender diverse adolescents and what role contextual factors may play in their mental health.

Given the high rates of adolescent referrals presenting with co-occurring psychological difficulties, further research in this area is urgently needed. In the cross-continental comparison studies between The Netherlands and North America, differences in psychological functioning in gender diverse youth were largely attributed to differences in peer relationship problems [11, 17, 18]. In The Netherlands, attitudes towards homosexuality have become more liberal since the 1960s [24], whereas in the USA, homosexuality was relatively less tolerated until the 1980s [25]. Therefore, it may not be surprising that greater acceptance of homosexuality was reported in The Netherlands compared to other Western countries [26]. In the current literature, no study has yet directly compared psychological functioning of gender diverse adolescents seeking input from gender services within Europe. The aims of this study were to assess psychological functioning and peer relationship problems in adolescents across four European specialist gender identity services (The Netherlands, Belgium, Switzerland, and UK).

## Methods

Four specialist gender identity clinics were taking part in this study: The Netherlands, Belgium, Switzerland, and the UK. All clinics were part of the Adolescent Gender Identity Research (AGIR) Group [27], a collaborative research group that have agreed to use the same assessment battery of tests that are both clinically useful and enable cross-clinic research. According to the Medical Research Involving Human Subjects Act (WMO), an official ethical approval of this study was not required.

1. The Center of Expertise on Gender Dysphoria, VU Medical Center Amsterdam, The Netherlands was established in 1988 at the University Medical Center in Utrecht. It moved to the VU Medical Center in The Netherlands in 2002. It is one of the longest standing gender services in the world. One other adult gender clinic is based in the north of the country. At the time of this study, all child and adolescent referrals were seen by the specialised child and adolescent professionals in the gender service in The Netherlands.
2. The Pediatric Gender Clinic, Ghent University Hospital, Ghent, Belgium, was founded in 2010. There are two other gender services in Liège and Brussels; however, Ghent is the only national clinic for young people.
3. The Department of Child and Adolescent Psychiatry, University Hospital of Psychiatry Zurich, Switzerland. This service was founded in 2009 and during the time of the study covered as catchment area the German speaking part of Switzerland.
4. The Gender Identity Development Service, Tavistock and Portman NHS Foundation Trust, London, United Kingdom, was established in 1989. The GIDS is a national clinic, with a satellite clinic in the north, based on Leeds and a number of other established outreach clinics in the Exeter, Dublin, and Wales.

The assessment protocol was broadly similar for each of the specialist gender clinics. In line with the international guidelines, the psychosocial assessment across all four clinics comprises of 3–6 appointments with one or two mental health professionals. Assessment appointments were offered around every 4–6 weeks. The mean duration of the diagnostic procedure was approximately 6–8 months. After the diagnostic work was finished, the applicant was discussed by the multidisciplinary team and the team together with the family decides whether the adolescent would benefit from further treatment, involving psychological and physical interventions.

### Participants

For this study, we included all adolescents aged 12–18 who were referred to one of the four European specialist gender clinics between January 2009 and December 2013. The total number of referrals combined was *n* = 1303. We analysed data from all referred adolescents who completed either the CBCL or the YSR at baseline. At the time that this information was gathered, none of the adolescents had started physical interventions. Data for *n* = 344 adolescents (26.4%) were not available due to not completing or not returning questionnaires. Therefore, they were excluded from the analyses. Therefore, the total number for the included sample was *n* = 959 (73.6% of the total number of adolescent referrals). In The Netherlands, a total number of 275 adolescents were referred between 2009 and 2013. Data were available for 252 young people (91.6%). For the UK clinic, 860 adolescents were referred in this time period and data were available for 610 young people (70.9%). For Belgium, 136 adolescents were referred and data for 71 young people were included in this study (52.2%). Switzerland had a total of 32 referrals over this time period, for which 26 were included in the sample (81.3%).

When comparing the included sample with the excluded sample, no significant differences were found with regard to birth-assigned gender, *χ* ^2^(1, 1075) = 1.8, *ρ* > 0.05. In this analysis, the excluded data were not available from Belgium or Switzerland. The excluded sample was found to be significantly older compared to the included sample, *F*(1, 1073) = 12.14, *ρ* < 0.05 (included *M* = 15.11, SD = 1.7 vs. excluded *M* = 16.15, SD = 1.2).

### Instruments

#### Background information

All referred adolescents were compared on several background measures: (1) assigned gender at birth; (2) age at assessment; (3) CBCL gender item 110; and (4) YSR gender item 110. On the CBCL and YSR, item 110 is specifically related to cross-gender identity (wishes to be the other sex). To avoid an artificial inflation in the calculation of behaviour problems on the CBCL and YSR, we set the value to “0” if item 110 was scored as 1 or 2 and the same was done for any other item if the parent identified gender-related issues. By taking this item out of the calculation, we used it as a measure for reporting gender dysphoric feelings.

#### Psychological functioning

The CBCL and YSR are standardised measures of behavioural and emotional problems in young people aged 6–18 years. The CBCL consists of 118 items and is completed by the parents. The YSR consists of 102 items and is completed by the young person. The answers were rated on a three point scale (0 = not true; 1 = sometimes true; and 2 = very true). It is possible to evaluate behaviour through the following scales: *Internalising*, *Externalising,* and the *Total Problem Scale*. In the present study, four dependent variables from the CBCL and YSR were used: (1) the mean total problem score, i.e., the sum of all items rated 1 or 2; (2) the mean score for internalising problems; (3) the mean score for externalising problems; and (4) and clinical range scores (> 90th percentile) for these three indices [15, 16]. For each country, a translation for CBCL and YSR was available [15, 16].

#### Peer relations

Following the procedure by Zucker et al. [28], a Peer Relation Scale (PRS) was created to measure the quality of peer relations. This scale was constructed by the following items from the CBCL and the YSR: “Does not get along with other kids” (Item 25), “Gets teased a lot” (Item 38), and “Not liked by other kids” (Item 48). In Zucker et al. [28], Cronbach’s alpha was 0.81 for this CBCL scale in a gender dysphoric adolescent sample. Likewise, a Peer Relation Scale was constructed from the corresponding YSR items, for which the Cronbach’s alpha was 0.63 [17].

### Statistical analyses

The data were scored locally for each of the four gender clinics for clinical and confidentiality purposes. All data were transported into a collective anonymised SPSS database. Analyses were done using the SPSS 22 software, using a significance of 5% (*α* = 0.05). To investigate if there were any differences in the demographic measures between the gender clinics, Chi-square tests and univariate tests were used. Demographic variables which were found to be significantly different were included as co-variates in further analyses.

Mean total problem scores and internalising and externalising scores were analysed by analysis of variance, using a 2 (Birth-assigned gender) × 4 (Clinic) AN(C)OVA and further post-hoc tests. Comparisons between the clinics in terms of psychological functioning (YSR, CBCL) and peer relations were analysed with use of ANCOVA’s tests, where age at referral, CBCL item 110, and YSR item 110 were used as co-variates in further analyses. In addition, differences in clinical range scores for the CBCL and YSR were analysed for clinic and assigned gender at birth using Chi-square tests.

## Results

### Demographic variables

Table 1 shows an overview of demographic data for each clinic. The sample showed that significantly, more birth-assigned girls were referred to the specialist gender services than birth-assigned boys, *χ* ^2^(1, 959) = 50.93, *ρ* < 0.01. Between-clinic differences were found, *χ* ^2^(1, 959) = 8.83, *ρ* < 0.05. The percentage of birth-assigned girls referred to the UK clinic was greater compared to the percentage of birth-assigned girls referred to The Netherlands, *Z*(1, 862) = − 2.82, *ρ* < 0.01.

**Table 1:** Background information as a function of clinic and assigned gender at birth

	Netherlands		Belgium		UK		Switzerland		
	*N* = 252		*N* = 71		*N* = 610		*N* = 26		
Assigned gender at birth									^a,^*
Assigned boys (*N*, %)	116	46.0%	24	33.8%	218	35.7%	11	42.3%	
Assigned girls (*N*, %)	136	54.0%	47	66.2%	392	64.3%	15	57.7%	
Age (in years)	14.30	2.18	14.34	1.65	15.54	1.28	15.38	1.20	^a,c,^*
Assigned boys (*M*, SD)	13.74	2.16	14.37	1.83	15.58	1.32	15.09	1.45	
Assigned girls (*M*, SD)	14.79	2.08	14.32	1.57	15.51	1.26	15.60	0.99	
CBCL item 110	1.80	0.49	1.71	0.62	1.90	0.36	1.91	0.29	^a,^*
Assigned boys (*M*, SD)	1.70	0.61	1.73	0.59	1.86	0.41	1.91	0.30	
Assigned girls (*M*, SD)	1.89	0.34	1.70	0.66	1.92	0.33	1.92	0.29	
YSR item 110	1.91	0.35	1.79	0.57	1.95	0.25	2.00	0.00	^a,b,c,^*
Assigned boys (*M*, SD)	1.84	0.46	1.67	0.72	1.94	0.26	2.00	0.00	
Assigned girls (*M*, SD)	1.97	0.20	1.91	0.43	1.96	0.24	2.00	0.00	

* p < 0.05

^a^ Significant difference between clinics

^b^ Significant difference between assigned genders at birth

^c^ Significant interaction birth-assigned gender × clinic

For age at referral, between-clinic differences were found which showed that adolescents from Switzerland and the UK were significantly older at referral (mean age = 15.39–15.54 years) compared to adolescents from The Netherlands and Belgium (mean age = 14.30–14.33 years), *F*(3, 958) = 42.18, *ρ* < 0.01. In addition, a significant interaction effect was found, *F*(3, 9) = 7.09, *ρ* < 0.01, showing that birth-assigned boys from both The Netherlands and Switzerland were on average significantly younger when referred to the gender services, whereas in the UK and Belgium, the birth-assigned girls were younger at referral.

For gender item 110 (*wishes to be of the opposite sex*), measured by the CBCL, a significant difference was found between clinics, *F*(3, 759) = 3.98 *ρ* < 0.01. Parents of adolescents from the UK clinic reported stronger cross-gender identification compared to parents of adolescents from The Netherlands and Belgium clinics, *F*(1, 701) = 8.34 *ρ* < 0.01. No effect was found for Swiss adolescents. No differences between birth-assigned genders were found.

For YSR item 110, a significant interaction effect was found, *F*(3, 769) = 3.21, *ρ* < 0.05. A significant main effect for clinic was found, *F*(3, 769) = 4.33, *ρ* < 0.01, revealing that adolescents from Belgium showed significantly less strong “wishes to be of the opposite sex” compared to all other clinics (UK, The Netherlands, and Switzerland). Furthermore, significant differences were found for birth-assigned gender, *F*(1, 769) = 5.76, *ρ* < 0.05, indicating that birth-assigned girls reported to have a stronger wish to be the other gender than birth-assigned boys.

The above demographics (age at referral, CBCL item 110, and YSR item 110) were used as co-variates in further analyses.

### Psychological functioning

#### Behavioural and emotional functioning

Table 2 shows the mean scores for the total problem scale, the internalising scale, and the externalising scale measured by the CBCL and YSR. A significant main effect between clinics was found on the CBCL total problem score, *F*(3, 759) = 3.91, *ρ* < 0.05. Post-hoc tests revealed that adolescents from The Netherlands showed significantly lower total problem scores compared to adolescents from the UK, *F*(1, 707) = 11.23, *ρ* < 0.01. No main effects were found for Belgium or Switzerland. In addition, no main effect for birth-assigned gender or interaction effects between birth-assigned gender and clinic was found.

**Table 2:** Ratings of behavioural problems for the three indices on the CBCL and YSR

	Netherlands			Belgium			UK			Switzerland			
	*N*	*M*	SD	*N*	*M*	SD	*N*	*M*	SD	*N*	*M*	SD	
CBCL													
Total problem score	223	44.25	27.62	35	48.86	28.56	479	51.24	29.78	23	52.96	23.73	^a,^*
Assigned boys	102	41.74	25.09	15	42.00	26.34	168	52.94	30.19	11	48.45	14.96	
Assigned girls	121	46.36	29.53	20	54.00	29.73	311	52.34	30.03	12	57.08	29.73	
Internalising	223	13.75	10.00	35	14.63	10.05	479	18.79	11.31	23	20.39	10.45	^a,^*
Assigned boys	102	12.36	9.33	15	12.27	10.53	168	17.17	10.82	11	20.36	7.49	
Assigned girls	121	14.93	10.43	20	16.40	9.55	311	19.66	11.49	12	20.42	12.94	
Externalising	223	10.04	8.95	35	12.57	9.44	479	11.48	9.51	23	11.91	9.30	
Assigned boys	102	9.01	8.03	15	10.47	8.13	168	12.15	9.61	11	9.45	5.45	
Assigned girls	121	10.91	9.61	20	14.15	10.23	311	11.12	9.45	12	14.17	11.60	
YSR													
Total problem score	219	45.67	22.05	37	60.49	23.91	489	67.14	29.95	25	54.68	18.39	^a,^*
Assigned boys	100	45.53	21.47	15	52.80	17.95	177	64.34	29.85	11	53.27	15.74	
Assigned girls	219	45.78	22.61	22	65.73	26.36	312	68.72	29.94	14	55.79	20.76	
Internalising	219	15.79	9.33	37	21.14	10.03	489	25.36	12.15	25	22.88	7.92	^a,^*
Assigned boys	100	16.09	9.27	15	19.33	7.61	177	24.12	11.93	11	21.55	7.65	
Assigned girls	219	15.54	9.41	22	22.36	11.40	312	26.06	12.24	14	23.93	8.25	
Externalising	219	14.76	7.24	37	18.05	9.63	489	18.58	9.58	25	16.92	6.16	^a,b,^*
Assigned boys	100	13.91	6.03	15	15.40	8.32	177	18.21	10.19	11	16.27	6.21	
Assigned girls	219	15.47	8.08	22	19.86	10.21	312	18.80	9.22	14	17.43	6.31	

*CBCL* Child Behavioural Checklist, *YSR* Youth Self-Report

* p < 0.05

^a^ Main effect for clinic

^b^ Main effect for assigned gender at birth

On the CBCL internalising problem score, a significant main effect was found between clinics, *F*(3, 759) = 7.7.25, *ρ* < 0.01. Adolescents from The Netherlands reported significantly less internalising problems compared to the UK clinic, *F*(1, 707) = 9.36, *ρ* < 0.01, and the Swiss clinic, *F*(1, 247) = 7.92, *ρ* < 0.01. No main effects were found for Belgium. No main effect for birth-assigned gender or interaction effect between birth-assigned gender and clinic was found.

For the CBCL externalising problem score, there were no significant differences found between clinics or birth-assigned gender. In addition, no interaction effects were found.

For the YSR, a significant main effect was found between clinics on the YSR total problem score, *F*(3, 769) = 22.06, *ρ* < 0.01. Adolescents from The Netherlands showed significantly lower total problem scores compared to both Belgium, *F*(1, 256) = 13.79, *ρ* < 0.01, and the UK, *F*(1, 715) = 89.55, *ρ* < 0.01. In addition, the Switzerland clinic showed significantly lower total problem scores compared to the UK clinic, *F*(1, 521) = 4.77, *ρ* < 0.05. No main effect for birth-assigned gender or interaction effect between gender and clinic was found.

On the YSR internalising problem score, a significant main effect was found between clinics, *F*(3, 769) = 25.01, *ρ* < 0.01. Adolescents from The Netherlands clinic showed significantly less internalising problems compared to adolescents from all the other gender clinics, Belgium [*F*(1, 256) = 10.23, *ρ* < 0.01], Switzerland [*F*(1, 245) = 13.50, *ρ* < 0.01], and the UK [*F*(1, 715) = 107.45, *ρ* < 0.01]. No main effect was found for birth-assigned gender. In addition, no interaction effect between gender and clinic was found.

For the YSR externalising problem score, between-clinic differences were found, *F*(3, 769) = 6.54, *ρ* < 0.01. Adolescents from The Netherlands showed less externalising problems compared to adolescents from the UK, *F*(1, 715) = 26.41, *ρ* < 0.01. No main effects were found for Belgium or Switzerland. Between-gender differences were found, *F*(3, 778) = 4.17, *ρ* < 0.05, indicating that externalising problem behaviour was more prevalent in birth-assigned girls compared to birth-assigned boys. No interaction effect between birth-assigned gender and clinic was found.

#### Clinical range

Table 3 shows the percentage of adolescents in each clinic, whose total problem score, the internalising score, and the externalising score fell in the clinical range on both the CBCL and the YSR (> 90th percentile).

**Table 3:** Clinical range on the three indices on the CBCL and YSR

	Netherlands		Belgium		UK		Switzerland		
	*N*	% (*N*)	*N*	% (*N*)	*N*	% (*N*)	*N*	% (*N*)	
CBCL									
Total problem score	224	38.8 (87)	35	54.3 (19)	484	51.7 (250)	24	37.5 (9)	^a,b,^*
Assigned boys	102	31.4 (32)	15	33.3 (5)	169	45.0 (76)	11	18.2 (2)	
Assigned girls	122	45.1 (69)	20	70.0 (14)	315	55.2 (174)	13	53.8 (7)	
Internalising	224	44.2 (99)	35	42.9 (15)	484	59.7 (289)	24	66.7 (16)	^a,^*
Assigned boys	102	39.2 (40)	15	40.0 (6)	169	56.2 (95)	11	81.8 (9)	^a,^*
Assigned girls	122	48.4 (59)	20	45.0 (9)	315	61.6 (194)	13	53.8 (7)	
Externalising	224	21.0 (47)	35	37.1 (13)	484	24.6 (119)	24	20.8 (5)	^b,^*
Assigned boys	102	11.8 (12)	15	20.0 (3)	169	23.7 (40)	11	9.1 (1)	
Assigned girls	122	28.7 (35)	20	50.0 (10)	315	25.2 (79)	13	30.8 (4)	
YSR									
Total problem score	220	20.5 (45)	37	43.2 (16)	469	46.4 (230)	26	15.4 (4)	^a,^*
Assigned boys	100	25.0 (25)	15	40.0 (6)	179	46.9 (84)	11	18.2 (2)	^a,^*
Assigned girls	120	16.7 (20)	22	45.5 (10)	317	46.1 (146)	15	13.3 (2)	^a,^*
Internalising	220	29.1 (64)	37	54.1 (20)	496	58.7 (291)	26	57.7 (15)	^a,b,^*
Assigned boys	100	39.0 (39)	15	60.0 (9)	179	66.5 (119)	11	63.6 (7)	^a,^*
Assigned girls	120	20.8 (25)	22	50.0 (11)	317	54.3 (172)	15	53.3 (8)	^a,^*
Externalising	220	22.3 (49)	37	29.7 (11)	496	38.3 (190)	26	26.9 (7)	^a,^*
Assigned boys	100	19.0 (19)	15	26.7 (4)	179	41.3 (74)	11	18.2 (2)	^a,^*
Assigned girls	120	25.0 (30)	22	31.8 (7)	317	36.6 (116)	15	33.3 (5)	

*CBCL* Child Behavioural Checklist, *YSR* Youth Self-Report

* p < 0.05

^a^ Main effect for clinic

^b^ Main effect for assigned gender at birth

On the CBCL total problem score, between-clinic differences were found, *χ* ^2^(3, 767) = 11.69, *ρ* = 0.01. A main effect was found between adolescents from the UK and The Netherlands, *χ* ^2^(1, 708) = 10.08, *ρ* < 0.01, indicating that a greater percentage of the UK adolescents scored in the clinical range compared to The Netherlands adolescents. No main effects were found for Belgium or Switzerland. In addition, differences in birth-assigned gender were found, showing a significantly greater percentage of birth-assigned girls scoring in the clinical range compared to birth-assigned boys (38.7% for birth-assigned boys vs. 53.2% for birth-assigned girls), *χ* ^2^(1, 767) = 15.28, *ρ* < 0.01. No interaction effect between gender and clinic was found.

For the CBCL internalising problem score, a significant difference was found between clinics, *χ* ^2^(3, 767) = 18.24, *ρ* = 0.01. Significant differences were found between adolescents from Switzerland and The Netherlands, *χ* ^2^(1, 248) = 4.40, *ρ* < 0.05, and the UK and The Netherlands, *χ* ^2^(1, 708) = 14.88, *ρ* < 0.01, indicating that a greater percentage of the adolescents from Switzerland and the UK scored in the clinical range compared to The Netherlands adolescents. No main effect was found for Belgium. In addition, no significant gender differences (50.5% for birth-assigned boys vs. 57.2% for birth-assigned girls) or interaction effects were found.

For the CBCL externalising problem score, no significant differences were found between clinics. A significant main effect was found between birth-assigned genders for the CBCL externalising problem score. A greater percentage of birth-assigned girls scored in the clinical range compared to birth-assigned boys (18.9% for birth-assigned boys vs. 27.3% for birth-assigned girls), *χ* ^2^(1, 766) = 7.09, *ρ* < 0.01. No interaction effects between assigned gender at birth or clinic were found.

For the YSR, between-clinic differences were found on the YSR total problem score, *χ* ^2^(3, 779) = 49.64, *ρ* < 0.01. Significantly less adolescents referred to The Netherlands had total problem scores in the clinical range compared to Belgium, *χ* ^2^(1, 257) = 9.09, *ρ* < 0.01, and the UK, *χ* ^2^(1, 716) = 43.27, *ρ* < 0.01. Similarly, significantly less adolescents from the Swiss clinic had problem scores in the clinical range compared to both the UK, *χ* ^2^(1, 522) = 9,59, *ρ* < 0.01, and Belgium, *χ* ^2^(1, 63) = 5.47, *ρ* < 0.05. No gender differences were found (38.4% for birth-assigned boys vs. 37.6% for birth-assigned girls). In addition, no interaction effect between birth-assigned gender and clinic was found.

For the YSR internalising score, several between-clinic differences were found, *χ* ^2^(3, 779) = 54.24, *ρ* < 0.01. Significantly less adolescents from The Netherlands clinic had problem scores in the internalising clinical range compared to all other gender clinics; Belgium *χ* ^2^(1, 257) = 8.97, *ρ* < 0.01, Switzerland, *χ* ^2^(1, 246) = 8.73, *ρ* < 0.01, and the UK, *χ* ^2^(1, 716) = 53.34, *ρ* < 0.01. In addition, significant differences were found between birth-assigned gender, indicating that a greater proportion of birth-assigned boys scored in the clinical range compared to birth-assigned girls (57.0% for assigned boys vs. 45.6% for assigned girls), *χ* ^2^(1, 779) = 9.78 *ρ* < 0.01. No interaction effects between birth-assigned gender and clinic were found.

Between-clinic differences were also found for the YSR externalising score, *χ* ^2^(3, 779) = 18.38, *ρ* < 0.01. Significantly more adolescents from the UK clinic scored in the externalising clinical range compared to adolescents from The Netherlands, *χ* ^2^(1, 716) = 17.62, *ρ* < 0.01. No gender differences (32.5% for birth-assigned boys vs. 33.3% for birth-assigned girls) or interaction effects between birth-assigned gender and clinic were found.

#### Peer relations

**Fig. 1:**
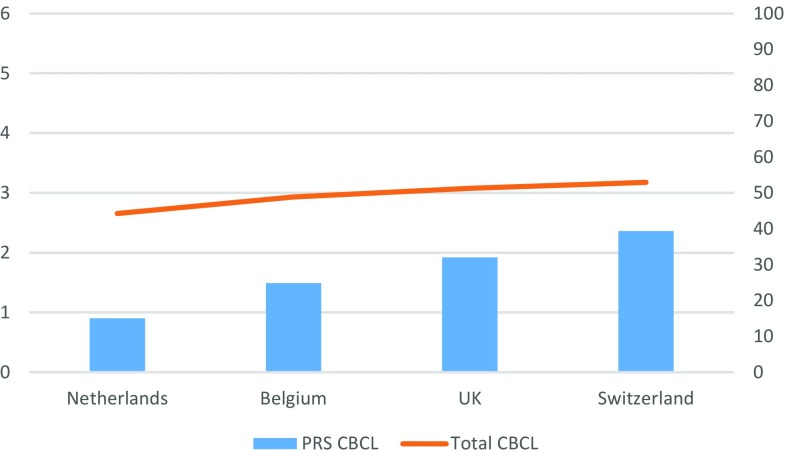
Mean scores on the CBCL Peer relations scale and the CBCL Total Problem Score per gender identity clinic

**Fig. 2:**
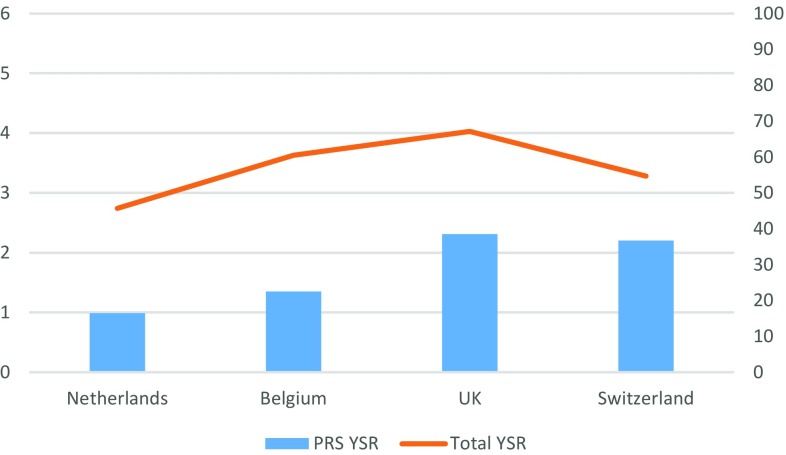
Mean scores on the YSR Peer relations scale and the YSR Total Problem Score per gender identity clinic

Figures 1, 2 show the results from the Peer Relation Scale (PRS) using both the CBCL and the YSR.

For the PRS measured by the CBCL, a significant main effect was found for clinic, *F*(3, 731) = 19.44 *ρ* < 0.01. Adolescents from the UK clinic, *F*(1, 702) = 49.74, *ρ* < 0.01, and from the Swiss clinic, *F*(1, 247) = 17.67, *ρ* < 0.01, reported significantly more peer relationship problems compared to the adolescents in The Netherlands. No main effect was found for Belgium. In addition, no significant gender differences or interaction effects between birth-assigned gender and clinic were found.

For the PRS measured by the YSR, a main effect for clinic was found, *F*(3, 761) = 35.45, *ρ* < 0.01. Adolescents from the UK [*F*(1, 713) = 88.44, *ρ* < 0.01] and Switzerland [*F*(1, 245) = 21.71, *ρ* < 0.01] reported more peer relationship problems compared to adolescents from The Netherlands. In addition, adolescents from the UK also reported significantly more peer relationship problems compared to the Belgian adolescents, *F*(1, 530) = 9.04, *ρ* < 0.01. Again, no gender differences or interaction effects were found.

## Discussion

Our study is the first cross-national comparison study examining psychological functioning in gender diverse adolescents across Europe, including four specialist gender identity clinics: The Netherlands, Belgium, Switzerland, and the UK. Standardised measures were used to assess behavioural and emotional functioning and peer relationship problems. Differences in emotional and behavioural problems and peer relationship problems were found across the four clinics. Overall, emotional and behavioural problems and peer relationship problems were most prevalent in adolescents from the UK, followed by Switzerland and Belgium. The least behavioural and emotional problems and peer relationship problems were reported by adolescents from The Netherlands. Across the four clinics, a similar pattern of gender differences was found. Birth-assigned girls showed more total behavioural problems and externalising problems in the clinical range, as reported by their parents. According to self-report, internalising problems in the clinical range were more prevalent in adolescent birth-assigned boys.

A rise in number of referrals, particularly in birth-assigned girls, is reported by gender clinics across the world [1, 8]. In line with this trend, the majority of adolescents referred to the gender clinics in Europe included in this study were assigned female at birth. A notable finding for the birth-assigned girls was that they reported more total problems and externalising problems in the clinical range than the birth-assigned boys. Different hypotheses are described in the literature contemplating why more birth-assigned girls present to gender services in adolescence. Aitken et al. [1] examined whether the severity of GD in birth-assigned girls had decreased over recent years, suspecting to see more “mild” cases coming forward. Another hypothesis was that “coming out” as trans is easier for birth-assigned girls than it is for birth-assigned boys [1], because birth-assigned boys tend to experience more stigmatisation for gender variance than birth-assigned girls [29]. However, to date, none of these hypotheses have yet provided sufficient support.

Alongside the higher prevalence of total behavioural problems, birth-assigned girls across the four gender clinics also reported a stronger desire “*to be of the opposite sex*” compared to the birth-assigned boys. Normative sex differences in pubertal onset, in which girls begin puberty on average at an earlier age than boys, may play a role in explaining these differences [1, 30]. Due to the earlier onset of puberty, birth-assigned girls start to develop secondary sex characteristics sooner which may lead to a longer period of distress related to their gender diverse feelings. The differences in pubertal onset could, to some extent, explain the higher problem scores and the more extreme desire to be of another gender as reported by the birth-assigned girls in this study.

Psychological problems and psychiatric comorbidity in gender diverse birth-assigned girls is a subject currently discussed in the literature [1, 8, 31]. An interesting finding in this study was that adolescent birth-assigned girls were reporting more externalising problems in the clinical range than the birth-assigned boys. No gender differences were previously found in externalising clinical range scores in gender diverse adolescents [11, 18]. However, adolescent birth-assigned girls did report a significantly higher externalising problem score, although not in the clinical range, than the birth-assigned boys in the most recent cross-clinic comparison study between Amsterdam and Toronto [17]. Similar to other studies, they found that birth-assigned boys showed significantly more internalising problems in the clinical range than did the birth-assigned girls, which was also in line with our findings [11, 17, 18]. As mentioned by de Vries et al. [17], does this mean that we see “*a general pattern of an* “*inversion*” *of internalising vs. externalising problems in relation to the sex*-*typical pattern of more internalising problems in girls and more externalizing problems in boys?*” More extensive research is needed to further investigate this phenomenon.

Although similarities were found across the four European gender clinics, there were also some differences, particularly in the prevalence of behavioural and emotional problems. On average, adolescents from the UK reported more problems compared to the other three gender services, both measured by parental- and self-report. In The Netherlands, adolescents reported considerably lower total problem scores and internalising problem scores, compared to the other three clinics. Adolescents from Belgium and Switzerland seemed to hold an in-between position.

Differences in behavioural and emotional functioning in adolescents across gender clinics were also found in the series of comparison studies between The Netherlands and North America. These studies showed that the Toronto adolescents had, on average, significantly more behavioural and emotional problems than the Amsterdam adolescents, both on the CBCL and on the YSR [17, 18]. It was suggested that these differences were more likely to be explained by a greater tolerance or acceptance of gender variance in Dutch culture than in North-American culture, rather than differences in demographic variables, health care access, or availability of GnRHa treatment, which were also analysed [17].

The gender diverse adolescents referred to the European clinics differed in various ways. First, the number of referrals was considerably different across the countries. The UK and The Netherlands received more referrals within the same time period compared to Switzerland and Belgium. The differences in number of referrals could also be related to the time, since treatment, such as puberty suppression and hormones, was offered in the different countries. The gender identity clinics in The Netherlands and the UK represent two of the longest established gender identity clinics in the world [32, 33], where resources for gender identity issues have been available for decades. Although the Swiss and Belgian gender clinics experience an increase in referrals, at time of this investigation, both specialist gender clinics were relatively new which can reflect why in that time period the number of adolescents seeking help for their gender identity issues was much lower.

Second, the age at which the adolescents were referred differed between clinics. There is no clear explanation why these differences occurred. The mean age for adolescents from the UK and Switzerland was higher compared to adolescents from The Netherlands and Belgium. In addition, an interaction effect was found, showing that birth-assigned boys in The Netherlands and Switzerland were presenting at younger age compared to the birth-assigned girls from those countries. In UK and Belgium, the opposite was found, showing that assigned girls were presenting at a younger age compared to the birth-assigned boys. From the previous literature, we would expect to see birth-assigned girls being referred at a later age [29]. For both the UK and Belgian clinics, the age differences between the birth-assigned boys and the birth-assigned girls were relatively small. In addition, both clinics had the highest proportion of birth-assigned girls referred to their clinics. With the increase in birth-assigned girls seeking professional support from gender services, can we foresee the development of a new trend, showing that birth-assigned girls are presenting to gender services at younger ages?

More broadly, differences in cultural and sociological factors across European countries may also play a role in the different clinical presentations between gender diverse adolescents. Although the four gender clinics are all based in the same continent, there are clear differences in child-well-being between European countries [34, 35]. Factors such as low SES (social-economic status), parental mental health, and poor social support were shown to have an impact on higher risk of adolescent mental health problems across Europe [36]. More in-depth research is needed to investigate whether these factors may have a specific impact on gender diverse young people.

It is well-documented in the literature that peer support and psychological well-being are strongly interlinked [37]. Gender diverse young people are generally at greater risk of peer victimisation and stigmatisation compared to the general population [38–40]. Bullying and isolation are commonly reported experiences from gender diverse adolescents [4, 6]. A study by Shiffman et al. 2016 [19] found that gender diverse birth-assigned boys reported more “social bullying” compared to the birth-assigned girls. In a Finnish study, with the majority of participants being assigned female at birth, *isolation* was found to be the strongest predictor for high psychopathology and a “confused” gender identity. This was likely to affect older referred adolescents [8].

In this study, no gender differences were found in terms of peer relationship problems. However, differences were found between clinics, showing that gender diverse adolescents from UK and Switzerland reported more peer relationship problems compared to adolescents from The Netherlands. Interestingly, there were more similarities between these two countries. First, both reported significantly more internalising problems compared to adolescents from The Netherlands, who reported the least peer relationship problems. Second, gender diverse adolescents from UK and Switzerland were older at referral compared to the other two clinics. Taking all these factors into account, could we hypothesize that older gender diverse adolescents, particularly those experiencing internalising problem behaviours, may be more prone to experience more peer relationship problems? Overall, our findings underline that there is a strong link between peer relationships and behavioural problems across the European countries. Peer relationships are a very important part of general adolescent development [41]. When working with gender diverse adolescents, clinicians should realise the importance of peer relationships for the adolescents’ mental health and try to ameliorate their peer relations.

A strength of this study was that the population sample included four national samples of specialist gender clinics within Europe. This also makes our sample one of the largest sample sizes on gender diverse adolescents that have been reported in the literature, thus far. One of the limitations of this study was that the population sizes differed considerably between the four countries. Although cross-national differences were found, higher numbers should be warranted for Belgium and Switzerland to make stronger statements about these countries. Second, although we had included several demographic variables, it is possible that other demographic variables that were not included in this study might have contributed to the discrepancies between the European clinics. Factors such as social class or living situation could have given a more nuanced explanation and should be included in future studies. In addition, in this study, we investigated psychological functioning and quality of peer relations separately, while the previous studies have used regression analyses to determine whether peer relationship problems would predict psychological problems. This analysis lacks in this particular study, as its main aim was to focus on the differences found between European clinics. While our findings give further evidence for the link between psychopathology and poor peer relationships, we would emphasize that future research should examine more broadly the impact of other cultural and societal factors, such as experienced stigma and internalised transphobia [42–44], which are likely to have an important relation with peer relationship difficulties and psychological problems in gender diverse young people.

In conclusion, this is the first cross-national comparison study examining psychological functioning in gender diverse adolescents across Europe. A similar pattern was found across European clinics, showing that the majority of referrals were assigned female at birth. Although there were differences found in psychological functioning across the four clinics, the most notable findings were the different clinical presentations between birth-assigned girls and birth-assigned boys. Future research should focus on gaining a better understanding of the different clinical presentations in gender diverse adolescents and investigating potential contextual factors. These findings should also inform professionals working with this population across centers in Europe, and elsewhere, whether there is a need to develop different clinical pathways for gender diverse birth-assigned boys and birth-assigned girls.
